# A Meta-Analysis of Interleukin-10 -592 Promoter Polymorphism Associated with Gastric Cancer Risk

**DOI:** 10.1371/journal.pone.0039868

**Published:** 2012-07-31

**Authors:** Huiping Xue, Ying-Chao Wang, Bing Lin, Jianfu An, Lu Chen, Jinxian Chen, Jing-Yuan Fang

**Affiliations:** 1 Division of Gastroenterology and Hepatology, Shanghai Jiao-Tong University School of Medicine Renji Hospital, Shanghai Institution of Digestive Disease and Key Laboratory of Gastroenterology & Hepatology, Ministry of Health Shanghai Jiao-Tong University, Shanghai, People's Republic of China; 2 Division of Nutrition, Zhongshan Hospital, Fudan University School of Medicine, Shanghai, People's Republic of China; 3 Bioinformatics Department, Shanghai Jiaotong University School of Medicine, Shanghai, People's Republic of China; 4 Department of General Surgery, Renji Hospital, Shanghai, People's Republic of China; The Chinese University of Hong Kong, Hong Kong

## Abstract

We aimed to explore the role of IL-10 -592 A/C SNP in the susceptibility to gastric cancer through a systematic review and meta-analysis. Each initially included article was scored for quality appraisal. 17 studies were eligible for the meta-analysis. We adopted the most probably appropriate genetic model (recessive model). Potential sources of heterogeneity were sought out via subgroup and sensitivity analyses, and publication biases were estimated. IL-10-592 AA genotype is associated with the reduced risk of developing gastric cancer among Asians and even apparently observed among Asians high quality subgroup, suggesting IL-10-592 AA genotype may seem to be more protective from overall gastric cancer in Asian populations. IL-10-592 AA genotype is also associated with the overall reduced gastric cancer susceptibility in persons with *H. pylori* infection compared with controls without *H. pylori* infection, suggesting IL-10-592 AA genotype may seem to be more protective from overall gastric cancer susceptibility in persons infected with *H. pylori*. IL-10-592 AA genotype is not associated with either pathologic subtypes (intestinal or diffuse) or anatomic subtypes (non-cardia or cardia) of gastric cancer susceptibility. Genotyping methods like direct sequencing should be highly advocated to be conducted in future well-designed high quality studies among different ethnicities or populations.

## Introduction

Worldwide gastric cancer incidence has decreased but its mortality still ranks second [1]–[3]. In Asia [4], especially China [5], gastric cancer constitutes the peak lethal malignancy. As is widely known, infectious, dietary, environmental, and genetic factors are implicated in gastric carcinogenesis, but only a minority of persons exposed to risk factors such as *Helicobacter pylori* (*H. pylori*) infection ultimately develop gastric cancer [6], which implies that host genetic susceptibility plays an important role in developing gastric cancer [7]–[9]. Such various susceptibilities could be partially explained by single nucleotide polymorphisms (SNPs) of susceptible genes [7]–[9]. During the pathogenesis from chronic gastritis to gastric cancer spawned by *H. pylori* infection, host activated neutrophils and mononuclear cells can produce not only proinflammatory cytokines such as interleukin (IL)-1β, IL-6, IL-8 and tumor necrosis factor (TNF)-αbut also anti-inflammatory cytokines like IL-10. Rivetingly, the level of IL-10 besides those of IL-1 and TNF-α could also be elevated in gastric mucosa infected with *H. pylori*.

IL-10, a potent pleiotropic cytokine, has the dual ability to immunosuppress or immunostimulate anti-cancer properties [10]. Interleukin-10 inhibits the production of pro-inflammatory cytokines by inhibition of T-helper 1 (Th1) lymphocytes and stimulation of B lymphocytes and Th2 lymphocytes and thus downregulates the inflammatory response [10]–[12]. The human IL-10 gene, located on chromosome 1q31–32, consists of five exons and four introns and one of polymorphisms is reported in its 5′ -flanking region at position -592 A/C SNP [13].

In 2003, El-Omar EM et al. [14] and Wu MS et al. [15] almost simultaneously published their separate study on IL-10-592 A/C SNP. Since then, researchers have consecutively reported associations of IL-10-592 A/C SNP with the susceptibility to gastric cancer, but with mixed or conflicting results [16]–[30]. Up to now, there have been two relevant published meta-analysis articles focusing on IL-10-592 A/C SNP [31], [32], but those two meta-analyses both failed to adopt the most likely appropriate genetic model, and thus the authentic values of statistical results could be compromised.

Accordingly, the aim of our meta-analysis was to shed more light, using the most appropriate genetic model, on the role of IL-10-592 A/C SNP in the risk of developing gastric cancer and to identify possible sources of heterogeneity among the eligible studies.

**Figure 1:**
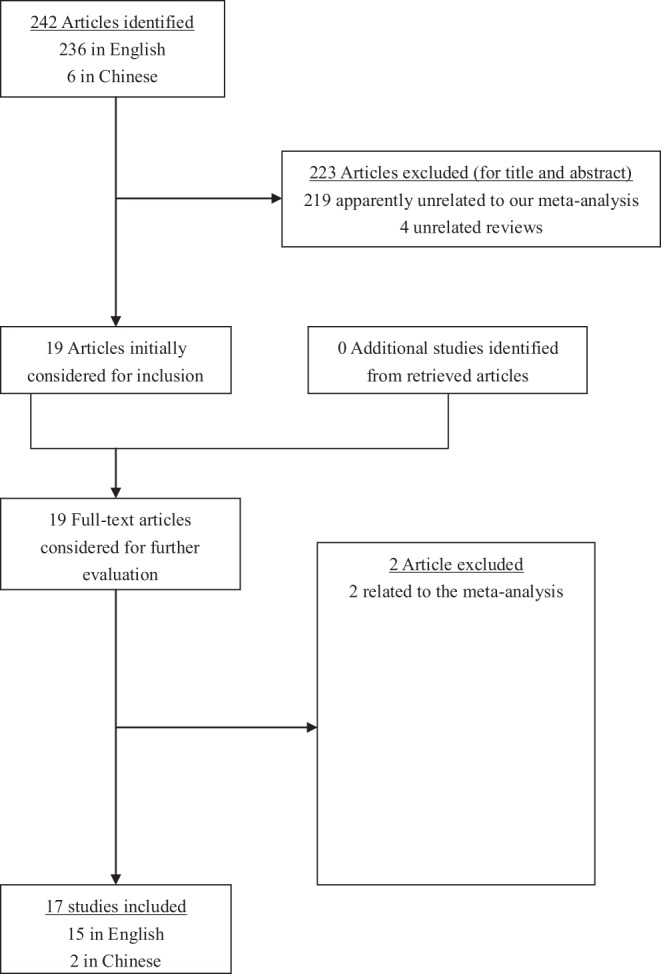
The flow chart of literature search and study selection.

**Table 1:** Study Characteristics of genotypes in gastric cancer cases and controls in the analysis of Interleukin-10 -592 Promoter Genetic Polymorphism.

First author	Year of publication	Quality assessment scores	Genotyping method	Total sample size	Number of controls	Number of cases	Study location	Ethnic group	P values for HWE	Controls, genotypes(n)			All Cases, genotypes(n)		
										CC	CA	AA	CC	CA	AA
El-Omar EM et al.^#∧^	2003	7.5	TaqMan	524	210	314	USA	Caucasians	0.427256638	127	70	13	178	101	35
Wu MS et al.	2003	7	Direct sequencing	450	230	220	China	Asians	0.231397685	20	83	127	27	105	88
Savage SA et al.	2004	5	ABI Genetic Analyzer	470	386	84	China	Asians	0.382599498	171	166	49	36	39	9
Alpízar-Alpízar W et al.	2005	6	Pyrosequencing	88	44	44	Costa Rica	Caucasians	0.761073904	18	21	5	21	20	3
Zambon CF et al.^∧^	2005	5	TaqMan	773	644	129	Italy	Caucasians	0.696436614	353	245	46	70	42	17
Lee JY et al.^+^	2005	5.5	RFLP	242	120	122	South Korea	Asians	0.059163504	7	60	53	8	62	52
Kamangar F et al.^∧^^¶^	2006	8	TaqMan	320	208	112	Finland	Caucasians	0.775545579	109	82	17	68	38	6
Sicinschi LA et al.^*^^¶^	2006	5.5	Pyrosequencing	550	369	181	Mexican	Latinos	0.376818571	98	176	95	51	90	40
Sugimoto M et al.^ *^^¶+^	2007	6.5	ASP	273	168	105	Japan	Asians	0.419149756	10	70	88	8	54	43
García-González MA et al.^#∧*^^¶^	2007	7.5	TaqMan	808	404	404	Spain	Caucasians	0.075218023	245	131	28	237	143	24
Crusius JB et al.^#∧^	2008	8.5	ABI real-time PCR	1359	1122	237	European	Caucasians	0.049349054	642	397	83	148	78	11
Deng WY et al.	2008	4	Direct sequencing	235	110	125	China	Asians	1.18833E-08	46	25	39	56	39	30
Kang JM et al.^*^^¶+^	2009	6.5	RFLP	665	332	333	Korea	Asians	0.591846755	41	145	146	34	157	142
Xiao H et al.	2009	6	RFLP	844	624	220	China	Asians	0.718880427	69	283	272	20	100	100
Ko KP et al.	2009	7	Snapshot	408	325	83	Korea	Asians	0.040647499	37	121	167	11	33	39
Con SA et al.^+^	2009	4.25	RFLP	243	191	52	Costa Rica	Latinos⊿	0.015843753	103	65	23	16	26	10
Liu J et al.	2011	6.5	RFLP	477	243	234	China	Asians	0.772829993	28	106	109	39	96	99

# Data of cardia-subtype gastric cancer were accessible; ^∧^ Data of noncardia-subtype gastric cancer were accessible; ^*^ Data of sporadic diffuse-subtype gastric cancer were accessible; ^¶^ Data of intestinal-subtype gastric cancer were accessible. ^+^Data of the status of *Helicobacter pylori* of gastric cancer were accessible. ⊿Here the ancestry of predominant participants in this study is annotated as Spanish ethnicity, which should be treated as Latinos rather than Caucasians [29]. RFLP: Restriction fragment length polymorphisms; TaqMan: 5′nuclease polymerase chain reaction assays; Pyrosequencing: a method of DNA sequencing (determining the order of nucleotides in DNA) based on the “sequencing by synthesis” principle. It differs from Sanger sequencing, in that it relies on the detection of pyrophosphate release on nucleotide incorporation, rather than chain termination with dideoxynucleotides; Direct sequencing: method of methylation analysis using bisulfite-treated DNA utilized PCR and standard dideoxynucleotide DNA sequencing to directly determine the nucleotides resistant to bisulfite conversion; ASP: the allele specific primer–polymerase chain reaction (ASP-PCR) method; Snapshot: the Snapshot assay which provides detection of certain SNPs.

**Figure 2:**
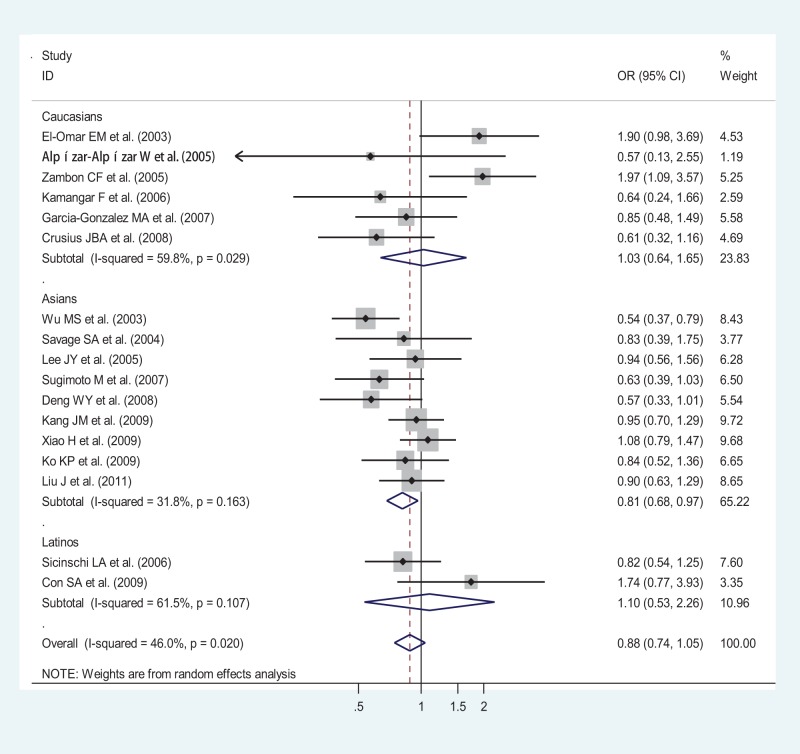
Odds ratios (ORs) for associations between IL-10 -592 A/C SNP and gastric cancer risk (AA vs CA-plus-CC) among different ethnicity populations, in order of increasing publication year, 2003–2011. Studies were entered into the meta-analysis sequentially by year of publication. The sizes of the squares indicate the relative weight of each study. Weights were derived from random-effects analysis. Bars, 95% confidence interval (CI).

**Figure 3:**
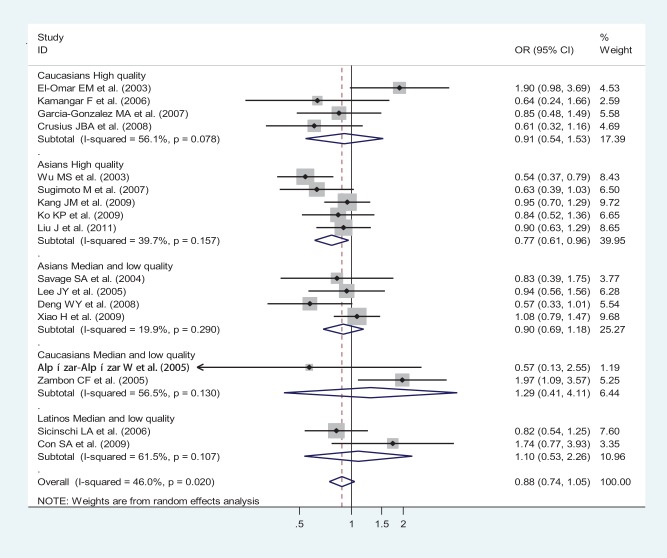
Odds ratios (ORs) for associations between IL-10 -592 A/C SNP and gastric cancer risk (AA vs CA-plus-CC) among different ethnicities based on high quality and median-and-low quality subgroup analysis. The sizes of the squares indicate the relative weight of each study. Bars, 95% confidence interval (CI).

**Figure 4:**
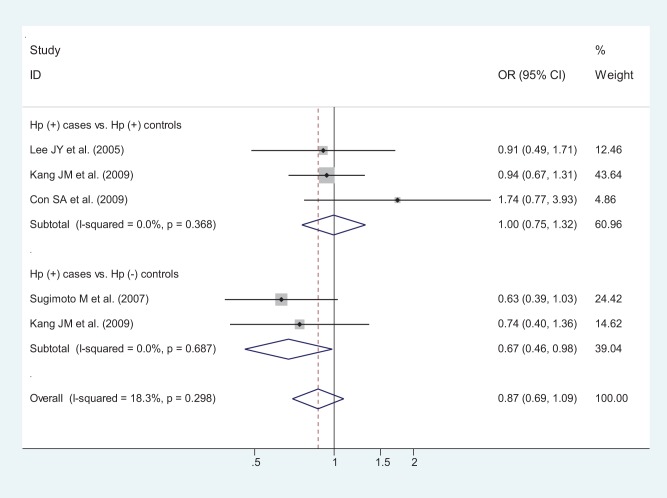
Odds ratios (ORs) for associations between IL-10 -592 A/C SNP and gastric cancer risk (AA vs CA-plus-CC) based on *H. pylori* infection status subgroup analysis. *H. pylori* positive cancer patients versus *H. pylori* negative controls and *H. pylori* positive cancer patients versus *H. pylori* positive controls, respectively. The sizes of the squares indicate the relative weight of each study. Bars, 95% confidence interval (CI).

**Figure 5:**
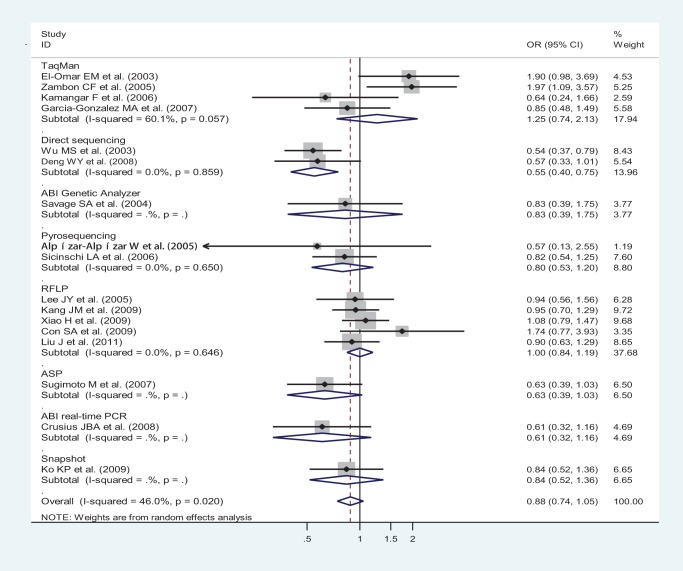
Odds ratios (ORs) for associations between IL-10 -592 A/C SNP and gastric cancer risk (AA vs CA-plus-CC) based on direct sequencing, TaqMan, ABI Genetic Analyzer, Pyrosequencing, RFLP, ASP, Snapshot, and ABI real-time PCR genotyping technique subgroup analysis. The sizes of the squares indicate the relative weight of each study. Bars, 95% confidence interval (CI).

**Figure 6:**
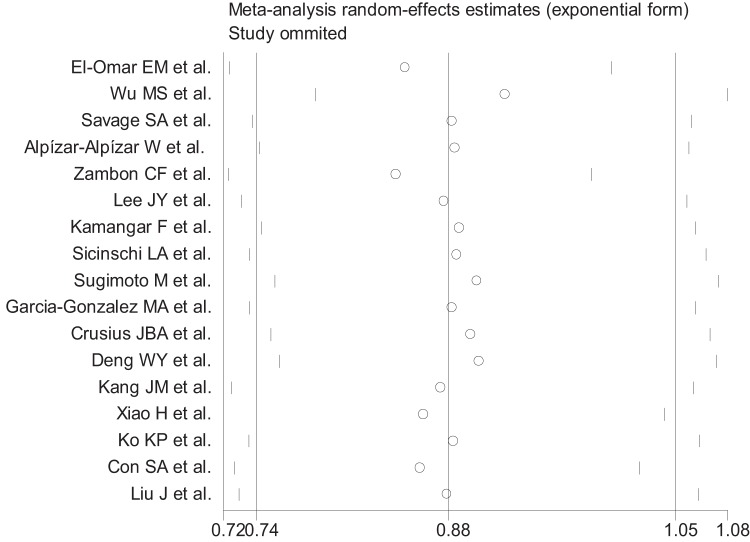
Influence analysis of the summary odds ratio coefficients on the association for the IL-10 -592AA genotype with gastric cancer risk. Results were computed by omitting each study (on the bottom) in turn. Bars, 95% confidence interval. Meta-analysis random-effects estimates (exponential form) were used.

**Figure 7:**
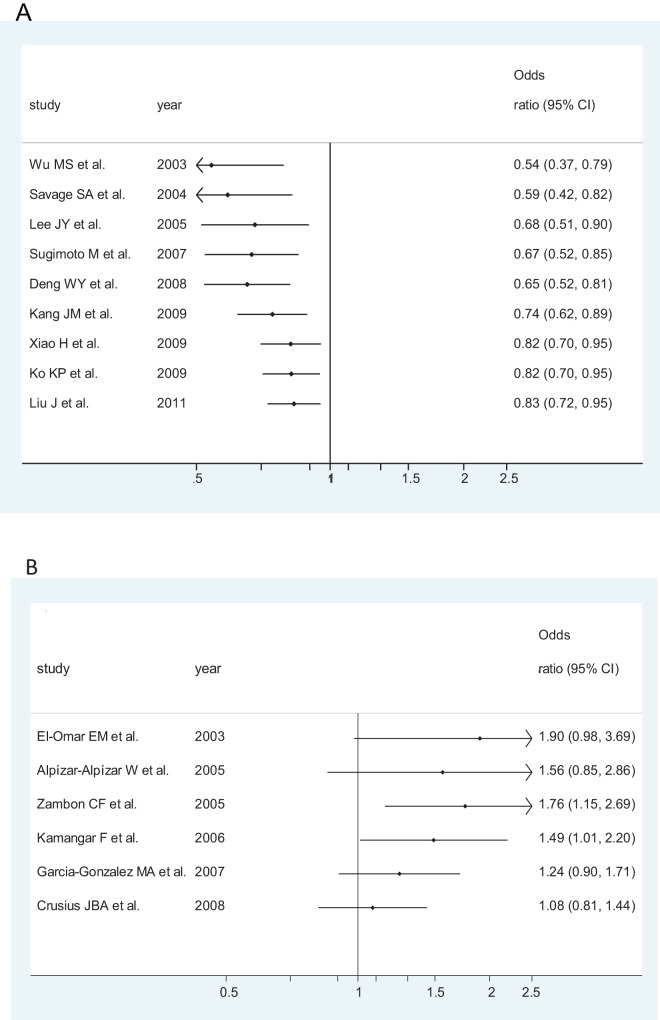
Cumulative meta-analysis of associations between the IL-10 -592AA genotype, as compared with the combined CA-plus-CC genotype, and gastric cancer risk among different ethnicity populations sorted by publication year and the total number of sample size. Horizontal line, the accumulation of estimates as each study was added rather than the estimate of a single study. A) among Asians; B) among Caucasians.

**Figure 8:**
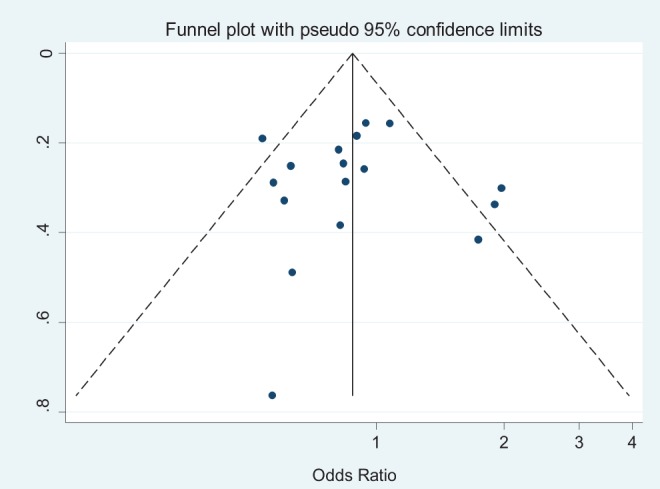
Funnel plot of publication bias for IL-10 -592 SNP (AA vs CA-plus-CC). Note: Funnel plot with pseudo 95% confidence limits was used.

## Materials and Methods

### Search Strategy

A systematic literature search was performed for articles regarding IL-10-592 A/C SNP associated with the risk of developing gastric cancer. The MEDLINE, EMBASE databases, Chinese National Knowledge Infrastructure (CNKI), Web of Science, and BIOSIS databases were used simultaneously with the combination of terms “Interleukin 10”, “IL-10”, “interleukin”, or “cytokine”; “gene”; “polymorphism”, “variant”, or “SNP”; and “gastric cancer”, “gastric carcinoma”, “diffuse gastric cancer” or “stomach cancer” from January 2000 to September 2011. The search was performed without any restriction on language. The scope of computerized literature search was expanded according to the reference lists of retrieved articles. The relevant original articles were also sought manually.

### Study Selection

Studies concerning the association of IL-10-592 A/C SNP with the risk of developing gastric cancer were included if the following conditions were met: (i) any study described the association of IL-10-592 A/C SNP with gastric cancer; (ii) any study reported the numbers of both controls and gastric cancer cases; (iii) results were expressed as odds ratio (OR) with 95% confidence intervals (CI); and (iv) studies were case-control or nested case-control ones.

### Methodological Quality Appraisal

To identify high-quality studies, we mainly adopted predefined criteria for Quality Appraisal [33], [34], [7]–[9]. The criteria cover credibility of controls, representativeness of cases, consolidation of gastric cancer, genotyping examination, and association assessment [7]–[9]. Methodological quality was independently assessed by two investigators (Y. Wang and B. LIN). Disagreements were resolved through discussion. Scores ranged from the lowest zero to the highest ten. Articles with the score lower than 6.5 were considered “low or moderate quality” ones, whereas those no lower than 6.5 were thought of as “high quality” ones.

### Data Extraction

The following data from each article were extracted: authors, year of publication, country, ethnicity of participants (categorized as Caucasians, Asians, Latinos, etc.), study design, source of controls, number of controls and of cases, genotyping method, distribution of age and gender, Lauren's classification (intestinal, diffuse, or mixed), and anatomical classification (cardia or non-cardia cancer).

The data were extracted and registered into two databases independently by two investigators (Y. Wang and B. Lin) who were blind to journal names, institutions or fund grants. Any discrepancy between these two investigators was resolved by the third investigator (H. Xue), who participated in the discussion with them and made an ultimate decision.

### Statistical Analysis

All statistical analyses were performed using STATA statistical software (Version 10.1, STATA Corp, College Station, TX). Two-sided Ps<0.05 were considered statistically significant. HWE in controls was calculated again in our meta-analysis. The chi-square goodness of fit was used to test deviation from HWE (significant at the 0.05 level). Odds ratios (OR) and 95% confidence intervals (95% CI) were employed to assess the strength of associations between IL-10-592 A/C SNP with gastric cancer risk. OR_1_, OR_2_, and OR_3_ regarding IL-10-592 A/C SNP were calculated for genotypes AA versus CC, CA versus CC, and AA versus CA, respectively.

The above pairwise differences were used to determine the most appropriate genetic model. If OR_1_ = OR_3_≠1 and OR_2_ = 1, then a recessive model is suggested. If OR_1_ = OR_2_ ≠1 and OR_3_ = 1, then a dominant model is implied. If OR_2_ = 1/OR_3_≠1 and OR_1_ = 1, then a complete overdominant model is suggested. If OR_1_>OR_2_>1 and OR_1_>OR_3_>1, or OR_1_<OR_2_<1 and OR_1_<OR_3_<1, then a codominant model is indicated [35]. If a dominant model was indicated, the original grouping was collapsed and the new group of A carriers (AA+CA) was compared with CC genotype; if a recessive model was suggested, AA was compared to the group of CC plus CA; if a complete overdominant model was implied, the group of AA plus CC was compared with CA; or if a codominant model was insinuated, AA was compared with CA and with CC, respectively.

The Q statistic was used to test for heterogeneity among the studies included in the meta-analysis. A fixed-effects model, using Mantel–Haenszel (M-H) method, was used to calculate the pooled ORs when homogeneity existed on the basis of Q-test p value no less than 0.1. By contrast, a random-effects model, using DerSimonian and Laird method (D+L), was utilized if there was heterogeneity based on Q-test p value less than 0.1. The significance of pooled ORs was tested by Z test (P<0.05 was considered significant).

Sensitivity analysis was performed, in which the meta-analysis estimates were computed after every one study being omitted in each turn.

Finally, publication bias was assessed by performing funnel plots qualitatively, and estimated by Begg's and Egger's tests quantitatively.

## Results

### Literature Search and Study Selection

After comprehensive searching, a total of 236 articles in English and 6 in Chinese were retrieved. In our meta-analysis were initially included altogether 17 studies [14]–[30] which catered to the inclusion criteria. Those 17 studies were preliminarily appropriate to the meta-analysis of the associations with gastric cancer regarding IL-10-592 A/C SNP.

Four studies [24], [25], [28], [29] were deviated from HWE. Generally speaking, any study that deviated from Hardy-Weinberg equilibrium through our calculation should have been removed; however, considering that the number of participants especially in the study [24] was large and given that sensitivity analyses would be conducted, we remained those four studies in our meta-analysis. Thus, 17 studies [14]–[30] with a total of 5730 controls and 2999 cases were ultimately eligible for the meta-analysis of IL-10-592 A/C SNP. The corresponding characteristics were seen in Table 1. The flow chart of literature search and study selection was illuminated in Figure 1.

### Overall Meta-analysis among Different Ethnicity Populations

OR_1_ (p value), OR_2_ (p value), and OR_3_ (p value) of IL-10-592 A/C SNP for overall ethnicities were 0.91 (p = 0.437), 1.00 (p = 0.950), and 0.87 (p = 0.030), respectively, potentially insinuating a recessive genetic model effect of putative protective A allele (OR_1_ = OR_3_<1 and OR_2_  = 1). Meanwhile, after ethnicity subgroup analysis, OR_1_ (p value), OR_2_ (p value), and OR_3_ (p value) of IL-10-592 A/C SNP among Asians were 0.82 (p = 0.080), 1.04 (p = 0.699), and 0.83 (p = 0.011), respectively, further suggesting a recessive genetic model effect of putative protective A allele (OR_1_ = OR_3_<1 and OR_2_  = 1). Thus, the genotype AA was compared with the combined genotype CA-plus-CC. As in figure 2, for overall gastric cancer no statistically significant finding could be observed among Caucasians and Latinos, respectively, whereas a statistically significant finding could be noted among Asians from the facts that the pooled ORs (95% CI, p value) were 1.03 (0.64–1.65, p = 0.913) and 1.10 (0.53–2.26, p = 0.802) for the former, respectively, but 0.81 (0.68–0.97, p = 0.019) for the latter.

### Further Subgroup Analysis

Specific data for IL-10-592 A/C SNP were classified in accordance with the quality appraisal scores, into high quality (scores no less than 6.5) and median-and-low quality (scores less than 6.5) subgroups among different ethnicities. A statistically significant finding was only witnessed in Asians high quality subgroup but not in Asians median-and-low quality subgroup, Caucasians high quality subgroup, Caucasians median-and-low quality subgroup, or Latinos median-and-low quality subgroup, given that the pooled ORs (95% CIs, p value) were 0.77 (0.61–0.96, p = 0.022), 0,90 (0.69–1.18, p = 0.437), 0.91 (0.54–1.53, p = 0.724), 1.29 (0.41–4.11, p = 0.664), or 1.10 (0.53–2.26, p = 0.802), respectively (Figure 3).

When gastric cancer was classified into non-cardia (or distal) and cardia subtypes, no statistically significant findings were found among non-cardia subtype or among cardia subtype on the grounds that the pooled ORs (95% CIs, p value) were 1.09 (0.57–2.11, p = 0.787) among non-cardia subtype and 0.78 (0.42–1.45, p = 0.432) among cardia subtype. In terms of pathology, gastric cancer could be classified into intestinal, diffuse, or mixed subtypes, and no statistically significant finding was observed in intestinal-subtype cancer or diffuse-subtype cancer, for the pooled ORs (95% CIs, p value) were 0.82 (0.64–1.06, p = 0.127) in the former and 0.89 (0.62–1.29, p = 0.546) in the latter.

In terms of *H. pylori* infection status, a statistically significant finding was found among *H. pylori* positive cancer patients in contrast as *H. pylori* negative controls, but no statistically significant finding was found among *H. pylori* positive cancer patients in contrast as *H. pylori* positive controls, for pooled ORs (95% CIs, p value) were 0.67 (0.46–0.98, p = 0.041) in the former and 1.00 (0.75–1.32, p = 0.978) in the latter (Figure 4).

And when genotyping techniques were considered, a statistically significant finding was noted in direct sequencing subgroup but not in any other genotyping technique subgroup. In the direct sequencing, TaqMan, ABI Genetic Analyzer, Pyrosequencing, RFLP, ASP, ABI real-time PCR, and Snapshot genotyping technique subgroups, pooled ORs (95% CIs, p value) were 0.55 (0.40–0.75, p = 0.000), 1.25 (0.74–2.13, p = 0.406), 0.83 (0.39–1.75, p = 0.618), 0.80 (0.53–1.20, p = 0.273), 1.00 (0.84–1.19, p = 0.997), 0.63 (0.39–1.03, p = 0.067), 0.61 (0.32–1.16, p = 0.132), and 0.84 (0.52–1.36, p = 0.475), respectively (Figure 5).

### Sensitivity Analysis

Meta-analyses were conducted repeatedly when each particular study had been removed. The results indicated that fixed-effects estimates and/or random-effects estimates before and after the deletion of each study were similar at large, suggesting high stability of the meta-analysis results. As shown in Figure 6, the most influencing single study on the overall pooled estimates seemed to be the study conducted by Wu et al.[15], the sensitivity analysis, however, indicated high stability of the results from the facts that the ORs (95% CI, p value) were 0.88 (0.74–1.05, p = 0.152) before the removal of that study and 0.92 (0.79–1.08, p = 0.332) after the removal of that study. In view of the study [24] conducted by Crusius JB et al. which is deviated from HWE, the ORs (95% CI, p value) were 0.86 (0.74–0.99, p = 0.037) before the removal of that study and 0.87 (0.75–1.01, p = 0.063) after the removal of that study for the all ethnicity, indicating moderate to high stability of the results. Similarly, for the other three studies with deviation from HWE [25], [28], [29], removal of the three studies one by one altered ORs (95% CI, p value) from 0.86 (0.74–1.00, p = 0.050), 0.86 (0.74–1.00, p = 0.050), and 0.86 (0.74–1.00, p = 0.050) to 0.86 (0.74–1.00, p = 0.050), 0.86 (0.74–1.00, p = 0.050), and 0.86 (0.74–1.00, p = 0.050), respectively, indicating high stability of the results. (The illustrating figures were omitted due to the length of paper).

### Cumulative Meta-analysis

Cumulative meta-analyses of IL-10-592 A/C SNP association were also conducted among Asians (Figure 7 part A) and among Caucasians (Figure 7 part B) via the assortment of total number of sample size. As shown in Figure 7 part A, the inclination toward significant reverse associations with overall gastric cancer, though somewhat undulated, was obviously seen among Asians, whereas in Figure 7 part B, the opposite tendency was observed among Caucasians.

### Publication Bias Analysis

Publication bias was preliminarily examined by funnel plots qualitatively and estimated by Begg's and Egger's tests quantitatively. Its funnel plot (Figure 8) showed that dots nearly symmetrically distributed, predominantly within pseudo 95% confidence limits. P values were 0.902 in Begg's test and 0.914 in Egger's test, separately, also suggesting no publication bias.

## Discussion

In our meta-analysis, a statistically significant finding could be noted with the overall reduced risk of gastric cancer among Asians but not among Caucasians or Latinos (AA vs CA-plus-CC); the opposite tendency toward the risk of gastric cancer could also be observed between Caucasians and Asians via cumulative meta-analysis sorted by publication time and the number of total samples. Thus, IL-10-592 AA genotype may seem to be more protective from overall gastric cancer susceptibility among Asians. To be sure, the different or even conflicting risk associations, if so, among different ethnicities should be further meticulously investigated and reconfirmed in the future.

Our further subgroup analyses also indicate that a statistically significant finding was only witnessed in Asians high quality subgroup but not in Asians median and low quality subgroup, Caucasians high quality subgroup, Caucasians median and low quality subgroup, or Latinos median and low quality subgroup (AA vs CA-plus-CC). It is natural that high-quality studies should be designed in the future so as to accurately explore the real associations between IL-10-592 AA genotype and gastric cancer susceptibility among different ethnicities.

Additionally, 5[14], [18], [20], [23], [24] out of 17 eligible studies were dealt with noncardia-subtype gastric cancer and 3 [14], [23], [24] with cardia-subtype gastric cancer. No statistically significant findings could be noted with either subtype (AA vs CA-plus-CC). 5 studies [20]-[22], [23], [26] in our meta-analysis were dealt with pathologically intestinal-subtype gastric cancer and 4 [21]-[23], [26] out of 17 studies were dealt with pathologically diffuse-subtype gastric cancer. No statistically significant finding could be noted in either intestinal-subtype or diffuse- subtype cancer (AA vs CA-plus-CC). As is known, cardia-subtype gastric cancer differs from noncardia-subtype gastric cancer in etiology, pathology, carcinogenesis, and/or prognosis [36]–[38], so is intestinal-subtype cancer versus diffuse-subtype cancer. It could be said that the indiscriminate combination of cardia-subtype and noncardia-subtype cases or of intestinal-subtype and diffuse-subtype cases in the majority of eligible studies may mask or at least underestimate the strength of the real associations [7]–[9].

Furthermore, it was reported that gastric cancer develops in those with *H. pylori* infection rather than in uninfected ones [39]. In our meta-analysis, a statistically significant reverse association with gastric cancer was found among *H. pylori* positive cancer patients in contrast as *H. pylori* negative controls, but no statistically significant finding was found among *H. pylori* positive cancer patients in contrast as *H. pylori* positive controls (AA vs CA-plus-CC), indicating that IL-10-592 AA genotype may seem to be more protective from overall gastric cancer susceptibility in persons infected with *H. pylori*. Certainly, the real association between *H pylori* infection and IL-10-592 AA genotype and gastric cancer susceptibility should be further meticulously investigated in the future.

With the advent of new genotyping technologies like seminested polymerase chain reaction, TaqMan allelic discrimination test, direct sequencing, the allele specific primer–polymerase chain reaction, pyrosequencing, Snapshot, or real-time PCR, we can anticipate an explosion of genetic association studies in the future. In our meta-analysis, a statistically significant reverse association with gastric cancer susceptibility was noted in direct sequencing genotyping technique subgroup but not in any other subgroup. We have previously mentioned that the most statistically significant result witnessed in direct sequencing technology in meta-analysis does not demonstrate that other technologies cannot be used. Nevertheless, the genotyping results by means of a novel genotyping technique should better be confirmed using direct sequencing. Under this circumstance, the novel genotyping technology can be seen as valid as direct sequencing [40]. Indeed, the sensitivity and specificity of those genotyping techniques need to be further explored so as to seek out the optimal approaches which could minimize the genotyping errors [7]–[9]. We advocate that direct sequencing should be further conducted in future studies.

Finally, the strength of our meta-analysis could be summarized as follows. We sought to find as many publications as we could by means of various searching approaches. We laid more emphasis on assessing biases across studies and pinpointing the potential sources of heterogeneity via subgroup analyses, and sensitivity analyses. We assessed the publication biases by means of Begg's and Egger's tests as well as funnel plot tests. Thus, we convince that the results of our meta-analysis, in essence, are sound and reliable.

Certainly, inevitable limitations could still be found in our meta-analysis. Firstly, the information extracted from the included studies is predominantly about genotypes associated with overall gastric cancer susceptibility, while less accessible is more important information regarding pathologic subtypes or anatomic subtypes of gastric cancer or regarding *H. pylori* infection status. Thus, the results of subgroup analyses in line with specific subtypes or *H. pylori* infection status should be considered with extreme caution. Secondly, given that merely published studies are included in our meta-analysis, publication bias could potentially occur, though no statistically significant publication bias is noted in our meta-analysis. Thirdly, moderate to severe heterogeneity could be witnessed across the included studies. Nonetheless, in an attempt to minimize the potential bias, we designed a rigorous protocol before conducting meta-analysis, and utilized explicit methods for literature search, study selection, data extraction, statistical analysis, genetic model adoption and sensitivity analysis [40], [41].

In conclusion, IL-10-592 AA genotype may seem to be more protective from overall gastric cancer susceptibility among Asians and may also seem to be more protective from overall gastric cancer susceptibility in persons infected with *H. pylori*. IL-10-592 AA genotype is not associated with either pathologic subtypes (intestinal or diffuse) or anatomic subtypes (non-cardia or cardia) of gastric cancer susceptibility in our meta-analysis. Such genotyping methods as direct sequencing should be highly advocated to be conducted in future well-designed high quality studies among different ethnicities or populations.
